# Identification, characterization and phylogenetic analysis of antifungal *Trichoderma* from tomato rhizosphere

**DOI:** 10.1186/s40064-016-3657-4

**Published:** 2016-11-09

**Authors:** Shalini Rai, Prem Lal Kashyap, Sudheer Kumar, Alok Kumar Srivastava, Pramod W. Ramteke

**Affiliations:** 1 ICAR-National Bureau of Agriculturally Important Microorganisms (NBAIM), Mau, Uttar Pradesh 275103 India; 2 ICAR-Indian Institute of Wheat and Barley Research (IIWBR), Regional Station, Flowerdale, Shimla, 171002 India; 3 ICAR-Indian Institute of Wheat and Barley Research (IIWBR), Karnal, Haryana 132001 India; 4 Sam Higginbottom Institute of Agriculture, Technology and Sciences (SHIATS), Allahabad, 211007 India

**Keywords:** Antagonism, Biocontrol, Diversity, Ergosterol, Tomato, *Trichoderma*

## Abstract

The use of *Trichoderma* isolates with efficient antagonistic activity represents a potentially effective and alternative disease management strategy to replace health hazardous chemical control. In this context, twenty isolates were obtained from tomato rhizosphere and evaluated by their antagonistic activity against four fungal pathogens (*Fusarium oxysporum* f. sp. *lycopersici*, *Alternaria alternata*, *Colletotrichum gloeosporoides* and *Rhizoctonia solani*). The production of extracellular cell wall degrading enzymes of tested isolates was also measured. All the isolates significantly reduced the mycelial growth of tested pathogens but the amount of growth reduction varied significantly as well. There was a positive correlation between the antagonistic capacity of *Trichoderma* isolates towards fungal pathogens and their lytic enzyme production. The *Trichoderma* isolates were initially sorted according to morphology and based on the translation elongation factor 1-α gene sequence similarity, the isolates were designated as *Trichoderma harzianum*, *T. koningii*, *T. asperellum*, *T. virens* and *T. viride*. PCA analysis explained 31.53, 61.95, 62.22 and 60.25% genetic variation among *Trichoderma* isolates based on RAPD, REP-, ERIC- and BOX element analysis, respectively. *ERG*-*1* gene, encoding a squalene epoxidase has been used for the first time for diversity analysis of antagonistic *Trichoderma* from tomato rhizosphere. Phylogenetic analysis of *ERG*-1 gene sequences revealed close relatedness of *ERG*-1sequences with earlier reported sequences of *Hypocrea lixii*, *T. arundinaceum* and *T. reesei.* However, *ERG*-1 gene also showed heterogeneity among some antagonistic isolates and indicated the possibility of occurrence of squalene epoxidase driven triterpene biosynthesis as an alternative biocontrol mechanism in *Trichoderma* species.

## Background

The genus *Trichoderma* has gained immense importance in past several decades due to its antagonistic ability against wide range of plant pathogens and growth promotion in crop plants. Some species of *Trichoderma* viz., *Trichoderma harzianum*, *T. viride*, *T. virens* and *T. koningii* are well known antagonists and are being utilized to control plant pathogens under field conditions (Solanki et al. 2011; Srivastava et al. 2012; Galarza et al. 2015). Promising *Trichoderma* isolates have different mechanisms or combination of direct parasitism, competition for nutrients, stimulators of plant health, or inducers of plant systemic resistance against various pathogens (Harman et al. 2004; Anees et al. 2010; Woo et al. 2014; Jain et al. 2015; Rai et al. 2016). A plethora of antagonistic *Trichoderma* isolates have been identified by several researchers from different places around the world, having history of varied climate, soil type, cropping system, etc., which differ in their innocuousness and efficacy as biocontrol agents (Sharma et al. 2009; Błaszczyk et al. 2011; Martínez-Medina et al. 2014; Galarza et al. 2015; El_Komy et al. 2015). Therefore, the site specific recommendations are being made according to the fitness potential of a particular isolate for higher efficacy and effectiveness. Despite the commercial successes of these biocontrol agents, the major limitations remain their restricted efficacy and inconsistency under field conditions. Consequently, more efficient *Trichoderma* isolates with high antagonistic potential capabilities are needed for successful biological control systems.

Due to the ecological importance of *Trichoderma* spp. and their application as a biocontrol agent in the field, it is important to understand their biodiversity. However, accurate species identification based on morphology is difficult due to the paucity and similarity of morphological characters and increasing numbers of morphologically cryptic species (Kullnig et al. 2001). This has already resulted in incorrect identification. In recent years, the usefulness of molecular markers such as random amplified polymorphic DNA (RAPD) and repetitive-element polymerase chain reaction (REP-PCR) in resolving species differences among microbial species are also well documented (Sharma et al. 2009; Solanki et al. 2013; Srivastava et al. 2014; Singh et al. 2014; Kashyap et al. 2015). RAPD utilized PCR to amplify DNA segments with single primer of arbitrary nucleotide sequence generating fragments by hybridizing with compatible regions of DNA and amplifying the regions where the primers are in correct orientation and appropriately spaced (100–2500 bp). However, REP-PCR uses oligonucelotide primers complementary to repetitive sequences dispersed throughout the genome. Using PCR, this method amplifies diverse regions of DNA flanked by the conserved repetitive sequences, leading to amplicon patterns specific for an individual bacterial and fungal strain. Three different families of repetitive sequences include: the 35–40 bp repetitive extragenic pallindromic (REP) sequence, the 124–127 bp enterobacterial repetitive intergenic consensus (ERIC) sequence and 154 bp BOX (composed of the box A, B and C subunits) element. These sequences appear to be located in distinct, intergenic positions around the genome elements (Mohapatra et al. 2007). Methods based on such repetitive elements have also been used for studying the diversity in the ecosystem, presenting the phylogenetic relationship between strains and discriminating between microorganisms those are genetically close to each other (Rai et al. 2015; Kashyap et al. 2016). Unfortunately, these methods have not been extensively used for the differentiation of *Trichoderma* spp. Since, species of *Trichoderma* are reported as the causal agent of green mould disease (Ospina-Giraldo et al. 1998), the understanding of the nature and diversity of *Trichoderma* is critical for its widespread use against phytopathogenic fungi as there could be the risk of unwanted disease on non-target hosts. Under such situations, it is valuable to establish patterns of gene flow, as well as to develop a fingerprint of *Trichoderma* isolates. Diversity studies have recently been undertaken to assess its ecological specialization. Several studies reported about a series of new isolates as well as new phylogenetic species of *Trichoderma* in a series of natural ecosystems (Zachow et al. 2009; Körmöczi et al. 2013). On the other hand, only a few studies were focusing on agricultural environments. However, the results of these studies demonstrated that besides the natural ecosystems, the investigation of agricultural soils also reveals important information about *Trichoderma* biodiversity. The practical impact of such studies is that the rhizosphere of agricultural soils may be an ideal source of beneficial strains with biocontrol potential. Based on these studies, we speculate that the species composition, distribution, and genetic structure of *Trichoderma* on the tomato rhizosphere may be different. The confirmation of the differences will help in revealing the biodiversity, origin, and evolutionary processes of *Trichoderma* under different biological niches.

Recent evidences indicated the importance of the sterol biosynthetic pathway in inducing plant defense-related gene expression in both the antagonistic fungus and the plant (Cardoza et al. 2011; Malmierca et al. 2013; Cardoza et al. 2014). The structural and functional analysis of genes involved in the synthesis of ergosterol especially intermediates, such as squalene could provide additional strategies to improve the ability of biocontrol of the *Trichoderma* strains. To best of the knowledge, there are no reports available on the diversity analysis of ergosterol producing antagonistic *Trichoderma* species using *ERG1* gene, encoding a squalene epoxidase, a key enzyme in the biosynthesis of triterpene derivatives (e.g. ergosterol) from tomato rhizosphere. Thus, to test above mentioned hypothesis, attempts have been made to investigate the species distribution of *Trichoderma* associated with tomato plants. The comparison of the genetic structure between antagonistic *Trichoderma* isolates was carried out by molecular (RAPD, REP, ERIC and BOX markers), and biochemical (production of cell wall degrading enzymes) markers. Sequencing based on the characterization of squalene epoxidase (*ERG*1) gene in antagonistic isolates was performed to get preliminary clues about the role of squalene epoxidase driven triterpene biosynthesis in biocontrol mechanisms of tested isolates.

## Methods

### Sampling and identification of *Trichoderma* isolates

Twenty isolates of *Trichoderma* were obtained from healthy tomato (*Solanum lycopersicum* cv. VL tamatar 4) rhizosphere (Table 1). Ten healthy plants (~55 days post transplanting) with their roots and rhizospheric soil were randomly sampled and immediately transported to the laboratory. The soil particles attached to roots were carefully collected after uprooting plants, stored at 4 °C and processed within 24 h of collection. Root adhered soil (10 g) was suspended in 90 ml of sterile distilled water and dilution plate technique was used for the isolation of *Trichoderma* spp. The suspensions from all samples were serially diluted (up to 10^−5^) and 100 µl of each dilution was added to sterile Petri dishes, in triplicates of each dilution, containing sterile Potato Dextrose Agar (PDA) medium. Streptomycin solution (1%) was added to the medium for preventing bacterial growth, before pouring into Petri plates. The plates were then incubated at 28 ± 1 °C. The isolates were characterized based on the monograph of Gams and Bissett (1998). For morphological analysis, isolates were grown on PDA at 28 ± 1 °C for 5–7 days. Radial growth was measured at 24 h intervals until colony covered the whole Petri dish. Growth rate was calculated as the 7 day average of mean daily growth (mm day^−1^). All micro morphological data were examined from cultures grown on PDA for 5 days at 28 ± 1 °C. Microscopic observations were done using trinocular microscope (Axio Imager M2 microscope, Carl Zeiss, Germany). For examination of conidial morphology, cultures were washed with sterile water and drops of the suspension were placed on microscope slides and mixed with lactophenol/cotton blue to stain the conidia. Length and width were measured for 30 conidia per isolate. Conidial morphology and size were recorded after 7 days of incubation.

**Table 1:** Identification, origin, NCBI Genebank accession numbers, cell wall-degrading enzymes and antagonistic effect of *Trichoderma* isolates from tomato rhizosphere against fungal plant pathogens

Code	Isolate (s)	Region	GenBank accession		Cell wall-degrading enzymes		Mycelia inhibition over control (%)			
			EF-1α	*ERG1*	Chitinase^x^	β-1,3 glucanase^y^	FOL	AA	CG	RS
UNT60	*Trichoderma harzianum*	U.S. Nagar, Uttarakhand	KF360991	KT989041	*40.00 ± 1.42^h^	83.67 ± 1.95^i^	58.54 ± 2.41 ^g^	58.89 ± 2.14^i^	59.80 ± 1.88 ^g^	53.30 ± 1.99 ^g^
UNT64	*T. harzianum*	U.S. Nagar, Uttarakhand	KF360992	KT989042	69.00 ± 3.54^b^	55.60 ± 0.85^l^	62.45 ± 1.56^f^	61.50 ± 1.91^h^	64.50 ± 2.11^cd^	62.78 ± 1.88^bcd^
UNT68	*T. harzianum*	U.S. Nagar, Uttarakhand	KF360993	KT989043	76.56 ± 3.95^a^	90.56 ± 0.88^g^	77.94 ± 2.21^a^	79.47 ± 1.88^a^	73.94 ± 1.88^a^	69.23 ± 1.35^a^
NAT69	*T. harzianum*	Nanital, Uttarakhand	KF360994	KT989044	53.33 ± 1.44^e^	53.33 ± 0.55^n^	57.65 ± 1.12^g^	53.98 ± 1.34^j^	59.50 ± 1.77^g^	54.80 ± 1.66^g^
NAT70	*T. harzianum*	Nanital, Uttarakhand	KF360995	KT989045	37.00 ± 1.20^i^	137.42 ± 0.65^e^	62.34 ± 1.21^f^	62.50 ± 1.99^gh^	60.89 ± 1.55^fg^	62.79 ± 1.64^bcd^
ALT73	*T. harzianum*	Almora, Uttarakhand	KF360996	KT989046	49.67 ± 2.01^f^	47.67 ± 0.44^p^	65.78 ± 1.54^e^	53.68 ± 1.86^j^	64.70 ± 1.66^cd^	63.65 ± 1.94^b^
DET89	*T. harzianum*	Dehradun, Uttarakhand	KF360997	KT989047	67.00 ± 3.93^b^	91.66 ± 1.99^g^	61.98 ± 1.34^f^	67.87 ± 1.54^d^	62.56 ± 1.35^e^	58.83 ± 1.51^e^
DET94	*T. harzianum*	Dehradun, Uttarakhand	KF360998	KT989048	69.00 ± 4.10^b^	171.66 ± 1.01^b^	74.35 ± 1.14^b^	65.50 ± 1.99^e^	68.89 ± 2.01^b^	67.89 ± 1.35^a^
HAT96	*T. harzianum*	Haridwar, Uttarakhand	KF360999	KT989049	73.60 ± 3.35^a^	63.26 ± 0.97^1^	68.95 ± 1.21^d^	63.45 ± 1.35 ^fg^	60.54 ± 1.35^fg^	61.11 ± 1.91^cd^
UNT38	*T. koningii*	U.S. Nagar, Uttarakhand	KF361001	KT989051	74.60 ± 3.55^a^	134.61 ± 1.06^f^	70.73 ± 1.12^c^	69.80 ± 1.36^c^	68.90 ± 1.55^b^	64.78 ± 1.35^b^
UNS63	*T. koningii*	U.S. Nagar, Uttarakhand	KF361002	KT989052	39.00 ± 1.99^h^	89.33 ± 1.04^h^	52.98 ± 1.33^i^	56.89 ± 1.25^i^	56.90 ± 1.97^h^	59.80 ± 2.11^de^
UNT13	*T. asperellum*	U.S. Nagar, Uttarakhand	KF361003	KT989053	56.56 ± 2.44^d^	51.26 ± 0.95^o^	55.80 ± 1.21^h^	61.89 ± 1.44^h^	62.80 ± 1.25^e^	59.80 ± 1.87^de^
UNT70	*T. asperellum*	U.S. Nagar, Uttarakhand	KF361004	KT989054	54.33 ± 1.66^e^	34.33 ± 0.65^q^	53.90 ± 1.44^i^	59.98 ± 0.99^h^	60.70 ± 1.35^fg^	62.89 ± 1.76^bcd^
UNS28	*T. virens*	U.S. Nagar, Uttarakhand	KF361005	KT989055	61.00 ± 1.32^c^	78.35 ± 0.78^j^	64.30 ± 1.31^e^	67.50 ± 1.34^d^	65.80 ± 1.97^c^	60.80 ± 1.54^cde^
UNS30	*T. virens*	U.S. Nagar, Uttarakhand	KF361006	KT989056	73.67 ± 2.12^a^	175.1 ± 1.20^a^	70.89 ± 1.21^c^	75.60 ± 1.65^b^	65.70 ± 1.54^c^	64.58 ± 1.62^b^
NAS46	*T. virens*	Nanital, Uttarakhand	KF361007	KT989057	44.00 ± 1.19^g^	54.22 ± 0.55^mn^	65.80 ± 1.33^e^	60.80 ± 1.21^hi^	59.80 ± 1.35^g^	57.50 ± 1.31^ef^
ALS47	*T. virens*	Almora, Uttarakhand	KF361008	KT989058	57.00 ± 2.37^d^	72.52 ± 0.72^k^	69.00 ± 1.54^d^	65.78 ± 1.35^e^	61.90 ± 1.67^e^	62.80 ± 1.15^bc^
UNT09	*T. viride*	U.S. Nagar, Uttarakhand	KF361011	KT989061	59.90 ± 1.15^c^	56.55 ± 0.96^l^	67.83 ± 1.34^d^	64.45 ± 1.44^ef^	60.85 ± 1.88^efg^	60.70 ± 1.25^cde^
DET02	*T. viride*	Dehradun, Uttarakhand	KF361012	KT989062	31.00 ± 1.25^j^	163.33 ± 1.22^c^	72.05 ± 1.68^c^	68.89 ± 1.55^cd^	64.44 ± 1.66^cd^	60.35 ± 1.35^cde^
NAT03	*T. viride*	Nanital, Uttarakhand	KF361013	KT989062	61.67 ± 2.03^c^	151.67 ± 1.10^d^	61.80 ± 1.34^f^	64.50 ± 1.39^ef^	59.48 ± 1.95^g^	60.45 ± 1.75^cde^

* Within columns, mean ± SE values with a common letter do not differ significantly (P < 0.05), according to DMRT test

^x^μmol of GlcNAc min^−1^ mg^−1^ protein

^y^nmol of glucose min^−1^ mg^−1^ protein

### Screening the antagonistic activity of *Trichoderma* isolates

In vitro antagonistic potential of the biocontrol agent was evaluated against *Fusarium oxysporum* f. sp. *lycopersici* (FOL), *Alternaria alternata* (AA), *Colletotrichum gloeosporoides* (CG) and *Rhizoctonia solani* (RS) through dual culture technique. For this, the pathogenic fungi were obtained from National Agriculturally Important Microorganisms Culture Collection (NAIMCC), NBAIM, Mau, Uttar Pradesh. After purification, the culture was maintained on PDA. The isolates were further screened for their antagonistic potential against the pathogen on PDA by measuring the relative growth rates as a function of the incubation period. Five mm mycelial discs taken from the margin of young vigorously growing 5-day-old culture of the antagonists and the pathogen was inoculated at the margin of the Petridish containing 20 ml sterilized PDA medium (opposite to each other). Observations were recorded up to 7 days of incubation (at 28 ± 1 °C). The treatments were replicated five times.

### Molecular characterization of antagonists

Total genomic DNA from fungus was extracted with cetyl-trimethylammonium bromide (CTAB) as described by Kumar et al. (2013a). Briefly, for each fungal isolates, fresh mycelium (~5 g) was dried on sterile blotter paper and was ground in liquid nitrogen to make a fine powder. This powder was taken in a centrifuge tube and 2× CTAB (hexadecyltrimethyl ammonium bromide) buffer (15 ml) was added in each tube separately. Extraction buffer contained (per 1 l) 2 g CTAB, 1 M Tris pH-8 (10 ml), 5 M NaCl (28 ml), 0.5 M EDTA (4 ml) with sterile distilled water (57 ml) and 1 ml β-mercaptoethanol. This was incubated in water bath at 65 °C for 30 min with intermittent shaking. The mixture was centrifuged at 13,000 rpm for 15 min at 4 °C to pellet the mycelium. Supernatant was taken into another Oakridge tube and an equal volume of phenol:chloroform:isoamyl alcohol (25:24:1) was added with slow inversion. The mixture was again centrifuged at 13,000 rpm for 15 min at 4 °C. The aqueous supernatant was taken in a fresh tube and added 0.6 volume isopropanol and was incubated at −20 °C overnight. After incubation, it was again centrifuged at 13,000 rpm for 20 min at 4 °C temperature. The supernatant was discarded and pellet was washed with 70% ethanol. The pellet was dissolved in 500 μl of TE buffer for the use in PCR and stored at −20 °C.

For the molecular identification of ergosterol producing isolates, *ERG1*F (5ʹ-CGCTCCGTGCTTCTTCTTCTC-3ʹ) and *EGR1*R (5ʹ-CTTCTTCTCTCCCGTCTCC-3ʹ) primers were used. The PCR reaction was carried out in a 25-μl reaction mixture containing the following: 10× PCR buffer, 50 ng DNA template, 2 mM MgCl_2_, 0.25 mM dNTP mixture and 0.25 μM each of primer, and one unit of *Taq* Polymerase (Bangalore Genie, India). Amplifications were performed in Thermal Cycler (G Storm GS4, Somerset, UK) under the following conditions: initial denaturation 5 min at 94 °C, 35 cycles of 45 s at 94 °C, 45 s at 58 °C, 1 min at 72 °C, with the final extension of 10 min at 72 °C.

Polymerase chain reaction (PCR) assay for translation elongation factor (*TEF*-*1a*) gene was conducted using primers TEF1-728 F and TEF1-986R (Al-Sadi et al. 2015). The PCR reactions were carried out in 25 μl reaction mixture containing 10× PCR buffer, 50 ng DNA template, 2 mM MgCl_2_, 0.25 mM dNTP mixture and 0.25 μM each of primer, and one unit of *Taq* Polymerase (Bangalore Genie, India). Thermocycling was run with the following settings: heating at 94 °C (5 min); then 35 cycles of 94 °C (30 s), 60 °C (30 s) and 72 °C (90 s). The final extension was done at 72 °C for 10 min.

Molecular characterization of *Trichoderma* isolates was assessed by rep-PCR using the BOXA1R, Rep1R-I, Rep2-I, ERIC-1R and ERIC-2F primers (Srivastava et al. 2014). All the PCR reactions were carried out in 25 μl reaction mixture containing 5× Gitschier buffer, 50 ng DNA template, 2 mM MgCl_2_, 0.25 mM dNTP mixture and 0.25 μM each of primer, and one unit of *Taq* Polymerase (Bangalore Genie, India). Thermal Cycler (G Storm GS4, Somerset, UK) was programmed as an initial denaturation at 94 °C for 5 min, 40 cycles of 94 °C for 1 min, 36 °C for 1 min and 72 °C for 2 min and a final extension at 72 °C for 10 min.

For RAPD assay, the DNA extracted from tested isolates was amplified with the RAPD primers using the five RAPD primer set (Bangalore Genei, India). The thermal profile used was initial denaturation at 94 °C for 5 min, followed by 35 cycles of denaturation step (94 °C for 1 min), annealing (47 °C, 1 min), extension (72 °C for 1.5 min), and a final extension step (72 °C for 7 min).

Amplified products were resolved in 2.0% agarose gels using 1× TAE buffer on a gel electrophoresis apparatus. Ethidium bromide (0.25 mg ml^−1^) was used as an intercalating agent. The gel was run at 2 V cm^−1^ of the length of gel till the bands resolved. The amplified bands, after separation on the gel, were visualized and documented using a gel documentation imaging system (Bio-Rad, USA).

### Statistical analysis

Experimental data for conidial morphology and growth rate were analyzed using Duncan’s multiple range test (DMRT). Standard errors were calculated for all mean values. All RAPD, ERIC, REP and BOX-PCR reactions were repeated to ensure validity of results. The presence or absence of individual, distinct, and reproducible bands was scored as ‘1’ for presence and ‘0’ for absence. Principle component analysis (PCA) was performed using XLSTAT software.

## Results

### Antagonistic activity of *Trichoderma* isolates

**Fig. 1:**
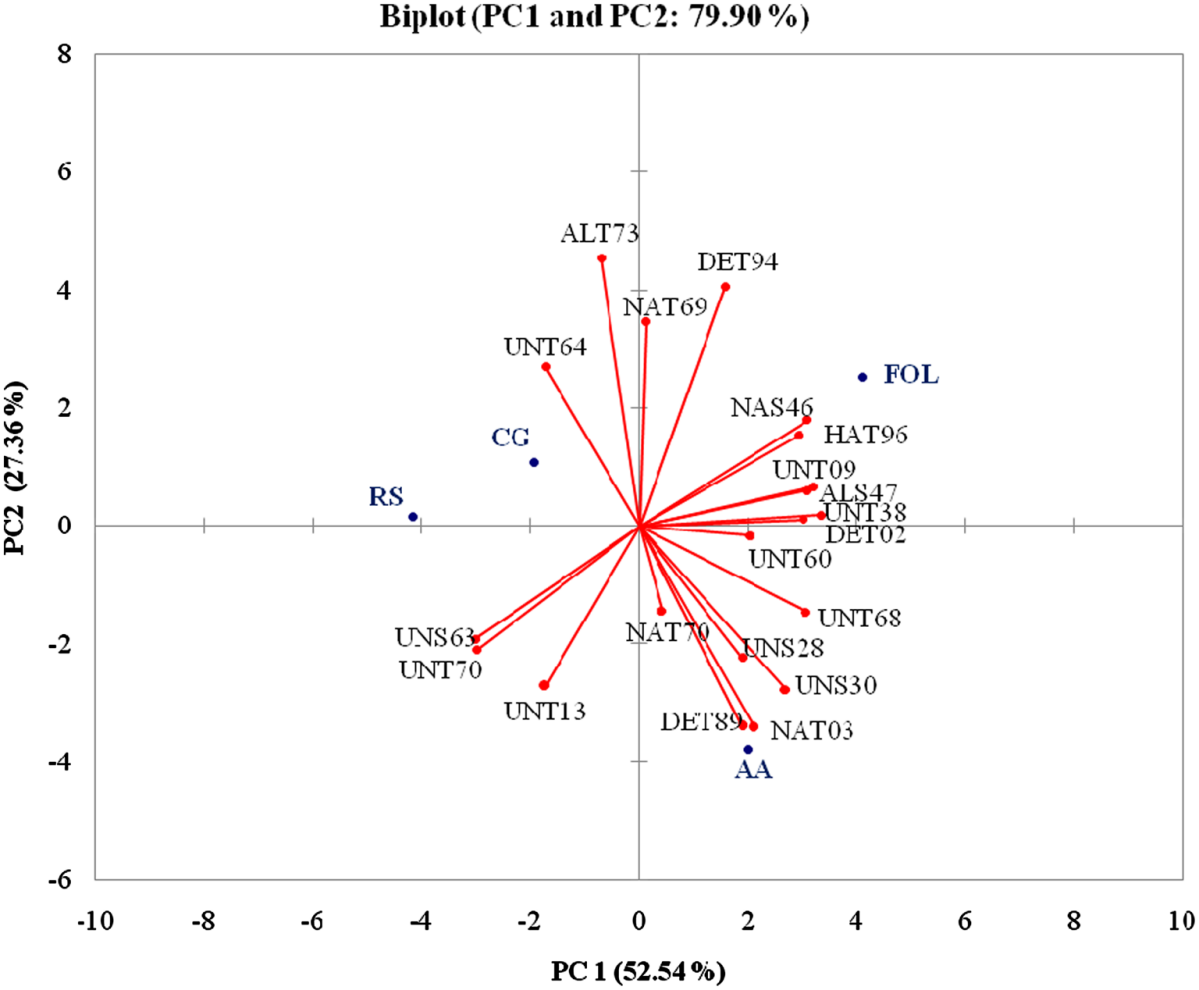
PCA biplot of in vitro dual culture assay showing antagonistic effect of twenty *Trichoderma* isolates against four fungal plant pathogens viz., *Fusarium oxysporum* f. sp. *lycopersici* (FOL), *Alternaria alternata* (AA), *Colletotrichum gloeosporoides* (CG) and *Rhizoctonia solani* (RS)

Antagonistic capabilities of the *Trichoderma* isolates were assessed by the growth inhibition of four fungal pathogens (FOL, AA, CG and RS) through the dual culture assay. In general, all the antagonistic isolates grew faster than pathogen. The interaction of biological control agents versus four different fungal pathogens showed significant differences in growth inhibition of the pathogen isolates (Table 1). Isolate UNT68 showed highest inhibition effect on the percent mycelia growth of FOL (77.94%), AA (79.47%), CG (73.94%) and RS (69.23). Contrarily, isolate UNT70, ALT73, UNS63 and UNT60 showed least percent mycelia growth of FOL (53.90%), AA (53.68%), CG (56.90%) and RS (53.30%), respectively. Most of the isolates showed per cent mycelium inhibition values ranged between 60 and 70% against pathogens. The interaction between pathogens and *Trichoderma* isolates were determined and illustrated by a biplot (Fig. 1). The first two principal component axis of the biplot accounted for 25.54% (PC1) and 27.36% (PC2) of the total variation of the pathogen–antagonist interaction. In this biplot, all the *Trichoderma* isolates were located very far from the origin of biplot, indicating strong antagonism of mycoparasitic isolates towards fungal plant pathogens. Eigen values of the first and second components were 10.508 and 5.471, respectively.

### Production of hydrolytic enzymes

All *Trichoderma* isolates used in the present study produced cell wall-degrading enzymes (chitinase and β-1, 3 glucanase). Data presented in Table 1 showed that all the mycoparasitic strains produced chitinase and β-1,3 glucanase in the range of 31.0–76.56 μmol GlcNAc min^−1^mg^−1^ protein and 47.67–175.1 nmol glucose min^−1^ mg^−1^ protein, respectively. Among all the isolates, maximum chitinase was produced by UNT68, HAT96, UNT38 and UNS30. Similarly, maximum β-1,3 glucanase production was observed in UNS30 followed by DET94 (Table 1). The lowest activities of chitinase (31.00 μmol GlcNAc min^−1^ mg^−1^ protein) and β-1,3-glucanases (51.56 nmol glucose min^−1^ mg^−1^ protein) were obtained for isolates DET02 and UNT13, respectively. However, most of the isolates showed moderate activities of both lytic enzymes (Table 1).

### Identification of antagonists

Distinct morphological differences were observed in 5 days old cultures of tested antagonistic isolates grown on PDA (Table 2). A perusal of data indicated that there was a significant difference in growth rate among isolates. Isolates UNT60, UNT68, NAT70, DET89, HAT96, UNT38, UNT13 and UNS30 grew faster (13.3 mm day^−1^) than other isolates. Less growth rate (11.4 mm day^−1^) was recorded in case of ALT73 and ALS47 isolates (Table 2). Ellipsoidal and sub-globose to globose conidia were noticed in thirteen isolates (UNT60, UNT64, UNT68, NAT69, NAT70, ALT73, DET89, DET94, HAT96, UNT38, UNS63, UNT09 and DET02). However, it was ellipsoidal and obovoid in rest of the eight isolates (UNT13, UNT70, UNS28, UNS30, NAS46, ALS47 and NAT03). Conidia colour varied from white to watery in all tested isolates. Fourteen isolates (UNT60, UNT64, UNT68, NAT70, DET89, DET94, HAT96, UNS63, UNT13, UNS28, UNS30, NAS46, UNT09 and DET02) showed conidiation concentric zone, while rings were also recorded in six isolates (NAT69, ALT73, UNT38, UNT70, ALS47 and NAT03). Phialides of most of the isolates were tending clustered in 2–3 whorls, but four isolate (NAT69, DET89, DET94 and NAT03) showed solitary disposition (Table 2). The phialides were nine-pin shaped and their size varied between 3.9–13.7 × 1.7–2.9 to 7.0–15.0 × 2.0–3.0 µm in seventeen isolates. However, globose and sigmoid or hooked phialides were also observed in two (UNT68 and ALT73) and one isolate (NAT03), respectively (Table 2).

**Table 2:** Morphological characteristics of *Trichoderma* isolates

Isolate	Colony				Mycelial		Conidia					Phialides		
	Growth rate (mm day^−1^)	Colour	Reverse colour	Edge	Form	Colour	Conidiation	Branching	Shape	Size (µm)	Colour	Shape	Size (µm)	Disposition
UNT60	*1.33 ± 0.17	Dark green	Creamish	Wavy	Floccose to Arachnoid	White	Concentric zones	Branched	ellipsodal, subglobose	1.5–3.4	Green	Nine-Pin shape	6–14 × 1.4–2.6	Tending clustered, 2–3 whorls
UNT64	1.32 ± 0.17	Dark green	Creamish	Wavy	Floccose to Arachnoid	Watery white	Concentric zones	Highly branched	ellipsodal, globose	1.3–3.6	Green	Nine-Pin shape	7–15 × 2–3	Tending clustered, 2–3 whorls
UNT68	1.33 ± 0.17	Light green	Light yellow	Wavy	Floccose to Arachnoid	Watery white	Concentric zones	Branched	ellipsodal, subglobose	1.6–3.0	Light Green	Globose	8–14 × 2–3	Tending clustered, 2–3 whorls
NAT69	1.16 ± 0.09	Dark green	Colourless	Wavy	Arachnoid	Watery white	Ring like zones	Highly branched	ellipsodal, subglobose	1.7–4.1	Green	Nine-Pin shape	6–14 × 1.4–3	Solitary
NAT70	1.33 ± 0.17	Dark green	Colourless	Smooth	Arachnoid	Watery white	Concentric zones	Highly branched	ellipsodal, subglobose	1.4–3.7	Green	Nine-Pin shape	5.6–14.8 × 2–3	Tending clustered, 2–3 whorls
ALT73	1.14 ± 0.12	Light green	Light yellow	Smooth	Floccose to Arachnoid	White	Ring like zones	Branched	ellipsodal, globose	1.3–3.3	Green	Globose	4.9–11.2 × 1.9–3	Tending clustered, 2–3 whorls
DET89	1.33 ± 0.17	Yellowish green	Light yellow	Wavy	Arachnoid	Watery white	Concentric zones	Branched	ellipsodal, globose	1.5–3.4	Light Green	Nine-Pin shape	6–15 × 1.4–2.8	Solitary
DET94	1.15 ± 0.09	Light green	Creamish	Smooth	Arachnoid	Watery white	Concentric zones	Highly branched	ellipsodal, subglobose	1.5–3.4	Light Green	Nine-Pin shape	5.9–15.2 × 1.9–2.8	Solitary
HAT96	1.33 ± 0.17	Dark green	Creamish	Smooth	Floccose to Arachnoid	White	Concentric zones	Moderately branched	ellipsodal, globose	1.5–3.6	Green	Nine-Pin shape	7–14.8 × 1.9–2.6	Tending clustered, 2–3 whorls
UNT38	1.33 ± 0.17	Light green	Light yellow	Wavy	Arachnoid	Watery white	Ring like zones	Highly branched	ellipsodal, globose	1.4–3.8	Green	Nine-Pin shape	6.2–10.2 × 2.2–2.9	Tending clustered, 2–3 whorls
UNS63	1.31 ± 0.16	Light green	Creamish	Wavy	Floccose	White	Concentric zones	Highly branched	ellipsodal, globose	1.5–3.9	Green	Nine-Pin shape	5.8–12.4 × 2.7–3.2	Tending clustered, 2–3 whorls
UNT13	1.33 ± 0.17	White to green	Light yellow	Wavy	Arachnoid	Watery white	Concentric zones	Highly branched	ellipsodal, obovoid	1.4–3.9	Dark Green	Nine-Pin shape	6.5–11.7 × 2.7–3.5	Tending clustered, 2–3 whorls
UNT70	1.32 ± 0.16	White to green	Light yellow	Smooth	Floccose to Arachnoid	White	Ring like zones	Moderately branched	ellipsodal, obovoid	1.5–3.8	Light Green	Nine-Pin shape	6.1–12.5 × 2.7–3	Tending clustered, 2–3 whorls
UNS28	1.31 ± 0.17	Light green	Creamish	Wavy	Floccose	Watery white	Concentric zones	Highly branched	ellipsodal, obovoid	1.4–3.6	Light Green	Nine-Pin shape	5.6–15 × 1.4–3	Tending clustered, 2–3 whorls
UNS30	1.33 ± 0.17	Light green	Light yellow	Wavy	Floccose	Watery white	Concentric zones	Highly branched	ellipsodal, obovoid	1.3–3.6	Green	Nine-Pin shape	6.8–14.4 × 2.2–3.2	Tending clustered, 2–3 whorls
NAS46	1.16 ± 0.09	Yellow to green	Light yellow	Wavy	Floccose to Arachnoid	Watery white	Concentric zones	Branched	ellipsodal, obovoid	1.4–3.5	Green	Nine-Pin shape	5.5–13.7 × 1.7–3.2	Tending clustered, 2–3 whorls
ALS47	1.14 ± 0.09	White to green	Light yellow	Wavy	Floccose	Watery white	Ring like zones	Branched	ellipsodal, obovoid	1.5–3.9	Light Green	Nine-Pin shape	4.5–12.0 × 1.7–3.0	Tending clustered, 2–3 whorls
UNT09	1.31 ± 0.16	Dark green	Creamish	Wavy	Arachnoid	White	Concentric zones	Moderately branched	ellipsodal, subglobose	1.3–3.9	Dark Green	Nine-Pin shape	3.9–13.7.0 × 1.7–2.9	Tending clustered, 2–3 whorls
DET02	1.32 ± 0.15	Yellow to green	Light yellow	Wavy	Floccose	White	Concentric zones	Moderately branched	ellipsodal, subglobose	1.5–3.7	Green	Nine-Pin shape	4.5–11.9 × 1.7–2.7	Tending clustered, 2–3 whorls
NAT03	1.16 ± 0.11	Light green	Light yellow	Smooth	Floccose	White	Ring like zones	Branched	ellipsodal, obovoid	1.5–3.7	Green	Sigmoid or hooked	5–12 × 2.2–2.7	Solitary

* Within columns, mean ± SE values with a common letter do not differ significantly (P < 0.05), according to DMRT test

**Fig. 2:**
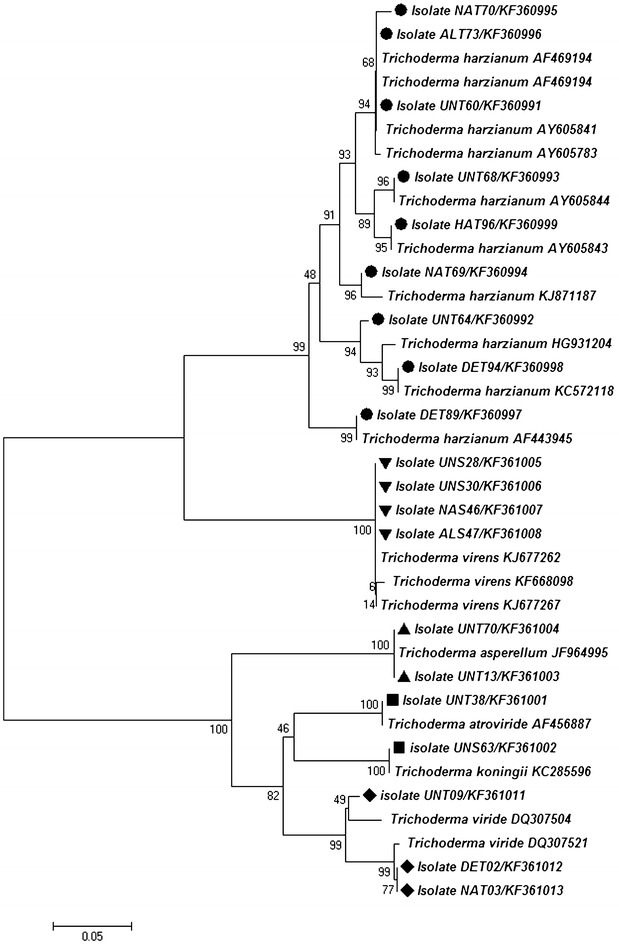
Neighbor joining tree (Kimura two-parameter distance) of twenty *Tef*-1a sequences of *Trichoderma* isolates from tomato rhizosphere. The *numbers* given over branches indicate bootstrap coefficient

Molecular identification based on sequences of *Tef*1gene confirmed that the isolates belonged to five different species viz., *T. harzianum* (UNT60, UNT64, UNT68, NAT69, NAT70, ALT73, DET89, DET94 and HAT96), *T. koningii* (UNT38 and UNS63), *T. asperellum* (UNT13 and UNT70), *T. virens* (UNS28, UNS30, NAS46 and ALS47) and *T. viride* (UNT09, DET02 and NAT03) (Table 1). The result of the phylogenetic analysis based on the *Tef*1 gene sequences of 20 *Trichoderma* isolates is shown in Fig. 2.

### RAPD-PCR analysis

**Fig. 3:**
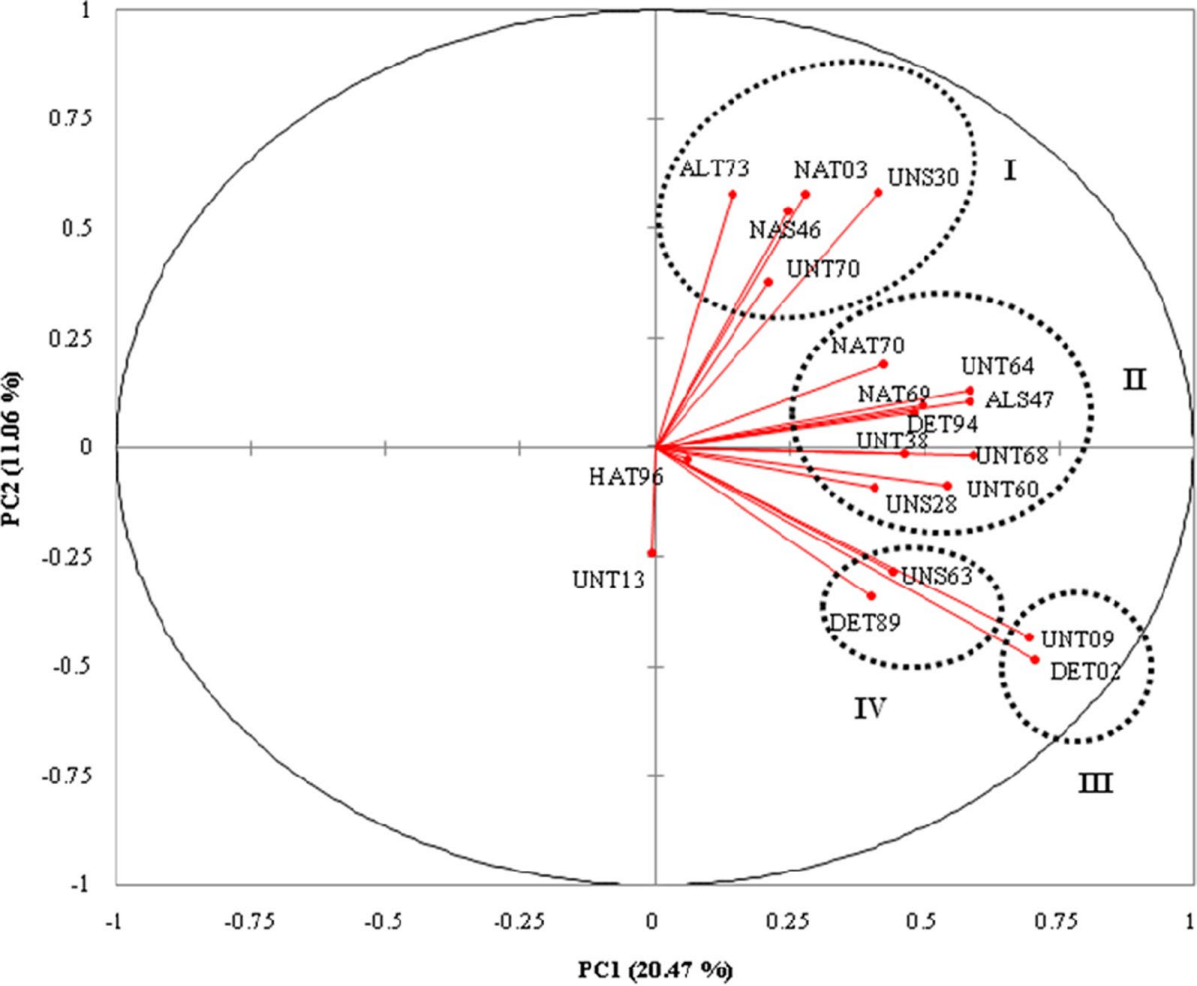
Principal component analysis score plot of twenty *Trichoderma* isolates based on RAPD-PCR data

Five primers viz., OPA-2 (TGCCGAGCTG), OPA-3 (AGTCAGCCAC), OPA-13 (CAGCACCCAC), OPA-15 (TTCCGAACCC) and OPA-18 (AGGTGACCGT) produced a total of 641 fragments among all the 20 isolates (Fig. 3). The size of RAPD fragments ranged 250–2500 bp. Principle component analysis (PCA) showed that RAPD markers explained 31.53% variation among *Trichoderma* isolates at genetic level (Fig. 3). PCA divided the 20 *Trichoderma* isolates in four clusters with pronounced separation of isolates. The first (PCA1) and second (PCA2) principal components were accounted for 20.47 and 11.06%, respectively. Two isolates occupied distinct position, UNT13 was far from the origin while HAT96 was near to the origin of biplot. Cluster I consisted of five isolates (ALT73, NAT03, UNS30, NAS46 and UNT70). However, cluster II comprised nine isolates (NAT70, UNT64, NAT69, ALS47, DET94, UNT38, UNT68, UNT60 and UNS28). Cluster III and IV contained two isolates each.

### BOX-PCR analysis

**Fig. 4:**
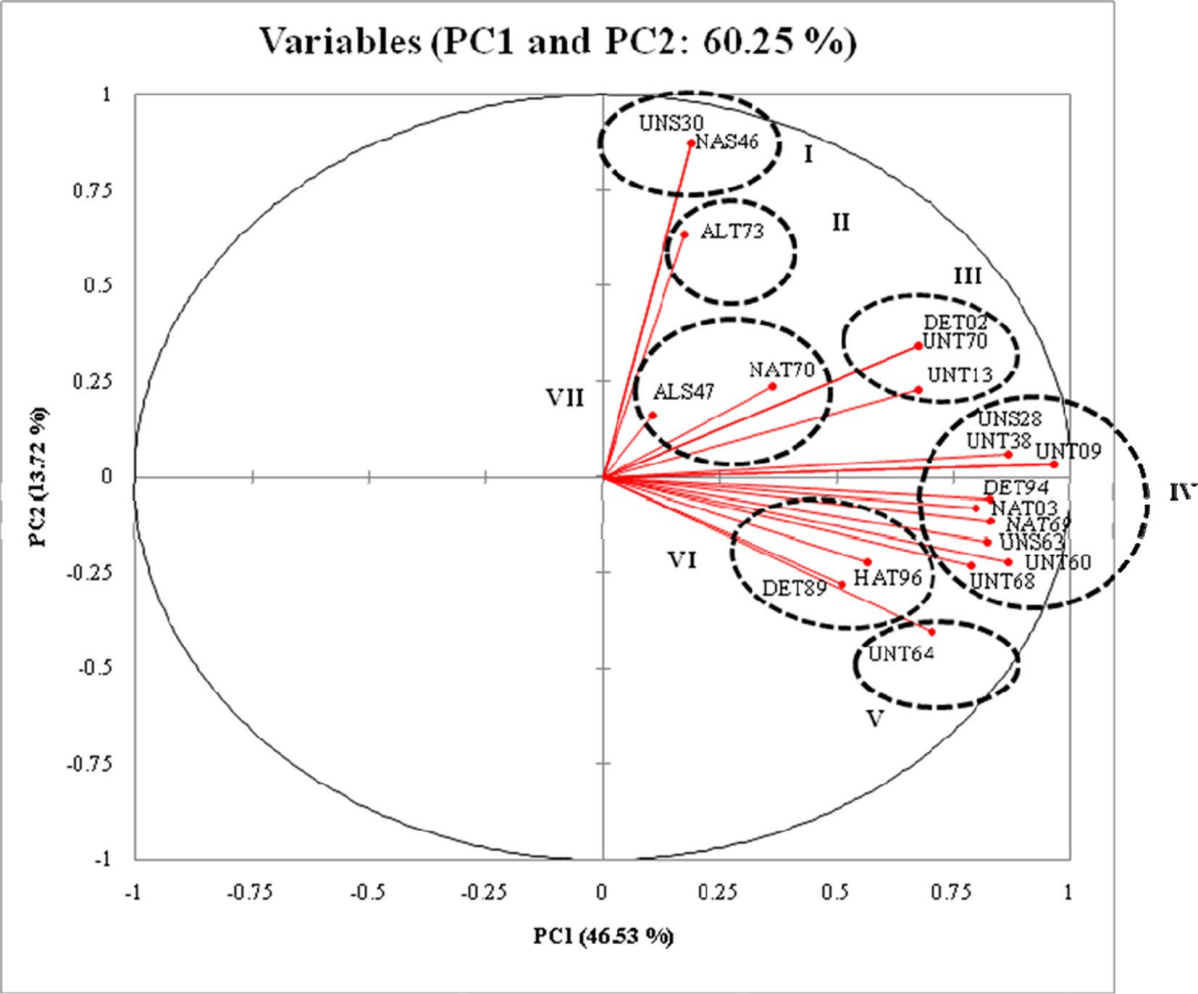
Principal component analysis score plot of twenty *Trichoderma* isolates based on BOX-PCR data

BOX-PCR banding pattern showed a total of 200 fragments in the range of 250–4000 bp. The results of PCA analysis based on first and second coordinates showed a maximum Eigen value of 9.306 and minimum value of 0.012 with a percentage variation of 46.53 and 13.72%, respectively (Fig. 4). PCA analysis revealed that nine isolates (UNS28, UNT38, UNT09, DET94, NAT03, NAT69, UNS63, UNT60 and UNT68) formed a major cluster (cluster IV), while three isolates (DET02, UNT70 and UNT13) were grouped in cluster II and two isolates were grouped in Cluster-I (UNS30 and NAS46), VI (DET89 and HAT96) and VII (ALS47 and NAT70).

### ERIC-PCR analysis

**Fig. 5:**
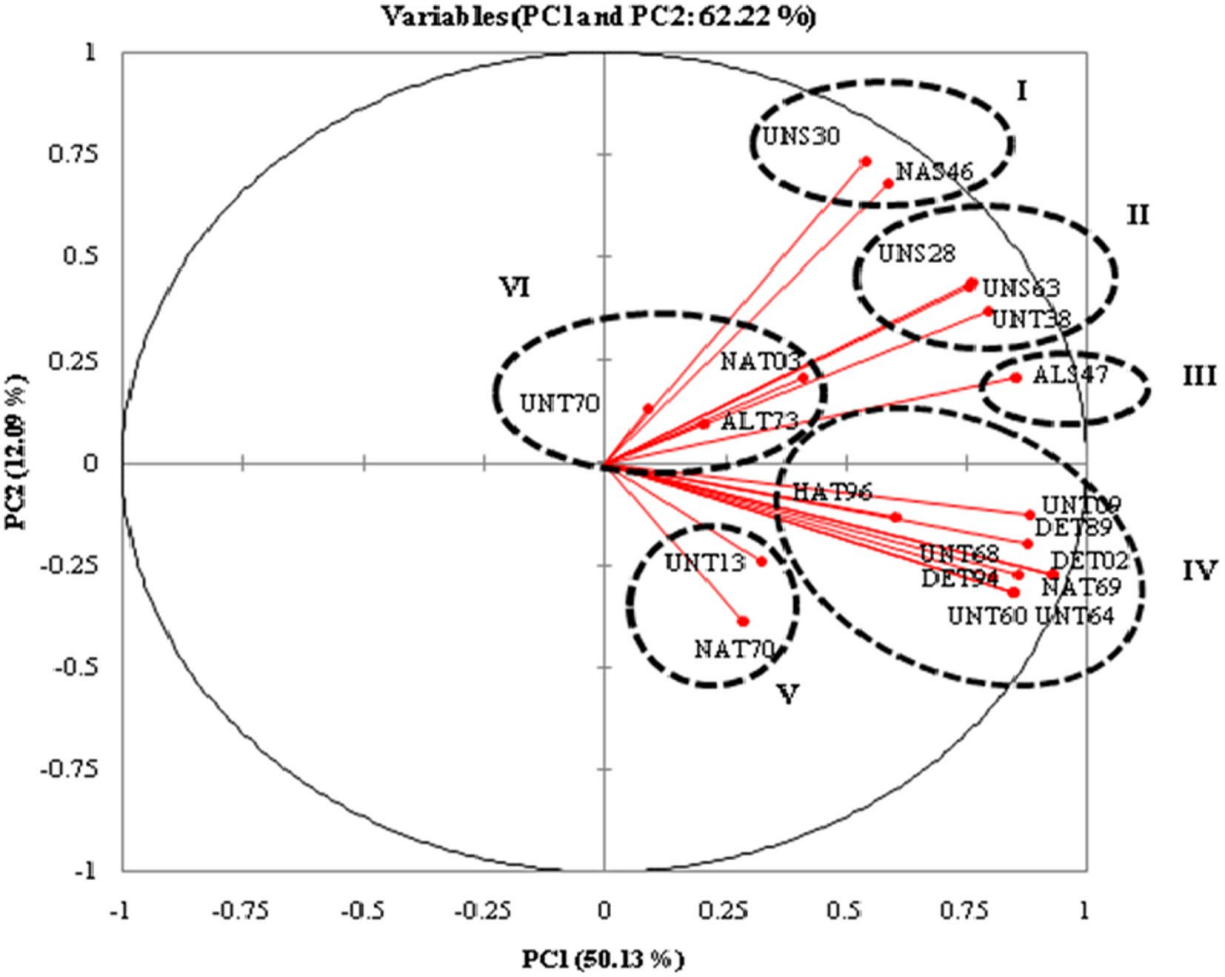
Principal component analysis score plot of twenty *Trichoderma* isolates based on ERIC-PCR data

The genetic discrimination among the 20 isolates was assessed using ERIC-PCR and a high level of variability in the banding pattern was obtained (Fig. 5). The number of bands in the amplification profile was 182, and their size was found to vary from 250 to 3000 bp among these isolates (Fig. 5). Principal component analysis (PCA) based on first and second coordinates showed a maximum Eigen value of 10.027 and minimum value of 0.01 with a percentage variation of 50.13 and 12.09, respectively (Fig. 5). A perusal of the PCA analysis revealed that eight isolates (HAT96, UNT68, DET94, UNT60, UNT64, NAT69, DET02, DET89 and UNT09) formed a major cluster (cluster IV), while three isolates were grouped in cluster II (UNS28, UNS63 and UNT38) and IV (UNT70, NAT03 and ALT73).

### REP-PCR analysis

**Fig. 6:**
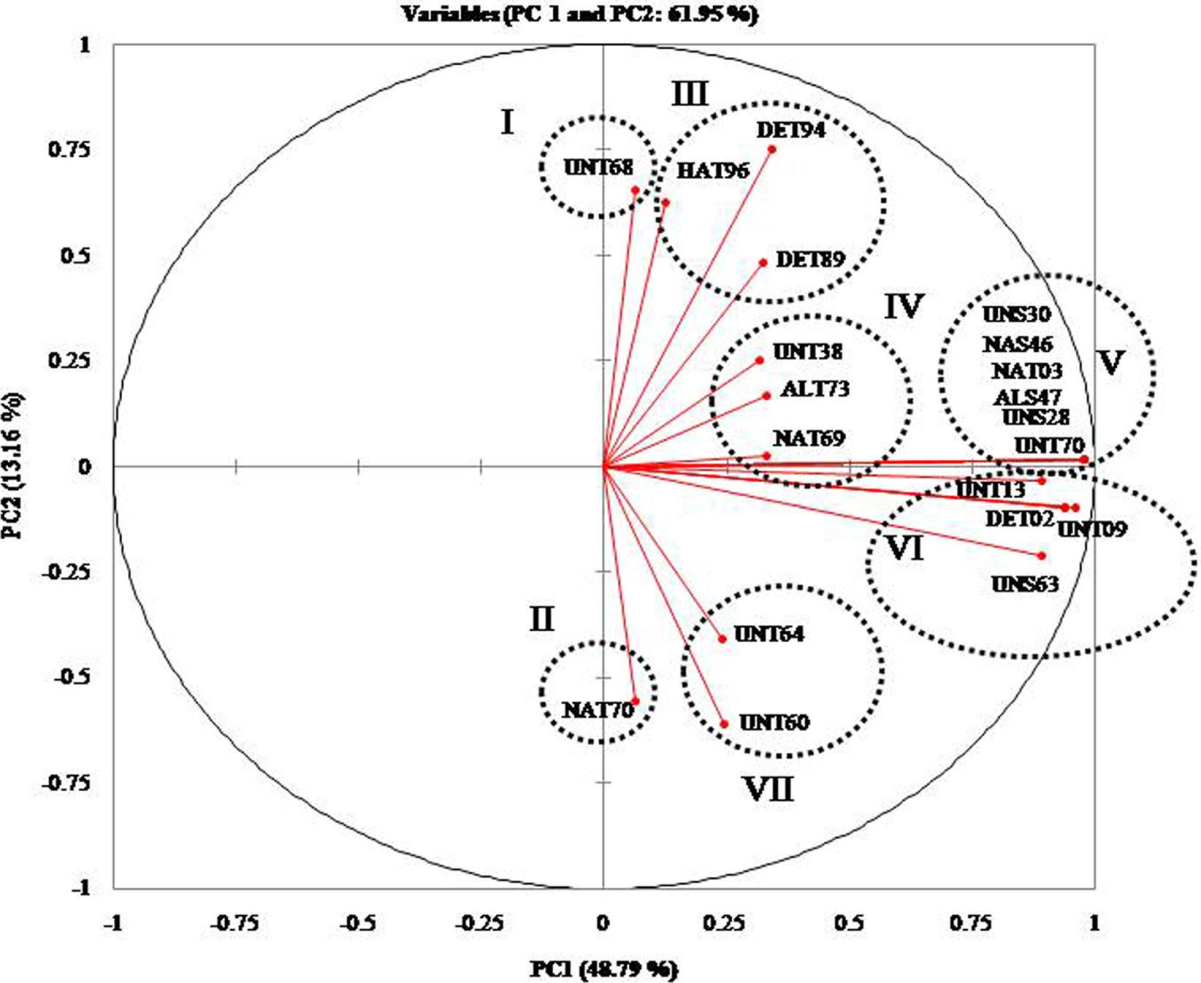
Principal component analysis score plot of twenty *Trichoderma* isolates based on REP-PCR data

The genetic discrimination among the 20 isolates was assessed using REP-PCR and a high level of variability in the banding pattern was obtained (Fig. 6). The number of bands in the amplification profile was 350, and their size was found to vary from 270 to 3000 bp among these isolates (Fig. 6). Principal component analysis (PCA) based on first and second coordinates showed a maximum Eigen value of 9.758 and minimum value of 0.017 with a percentage variation of 48.71 and 13.16, respectively (Fig. 6). A perusal of the PCA analysis revealed that six isolates (UNS30, NAS46, NAT03, ALS47, UNS28 and UNT70) formed a major cluster (cluster V), while four isolates were grouped in cluster VI (UNT13, DET02, UNT09 and UNS63).

### *ERG*1 sequencing and phylogenetic analysis

**Fig. 7:**
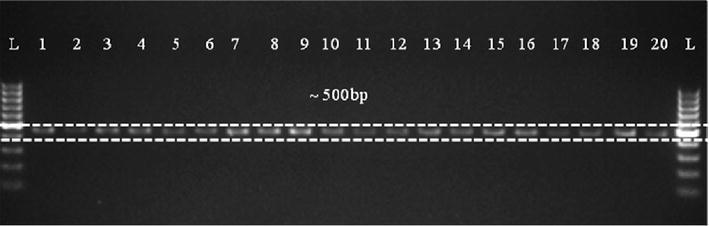
PCR amplification of squalene epoxidase (*ERG*1) gene, showing ~500 bp amplicon in *Trichoderma* isolates having distinct geographical lineages. *Lanes 1*–*20* are different *Trichoderma* isolates as mentioned in Table 1. *L* is a 100-bp DNA marker

**Fig. 8:**
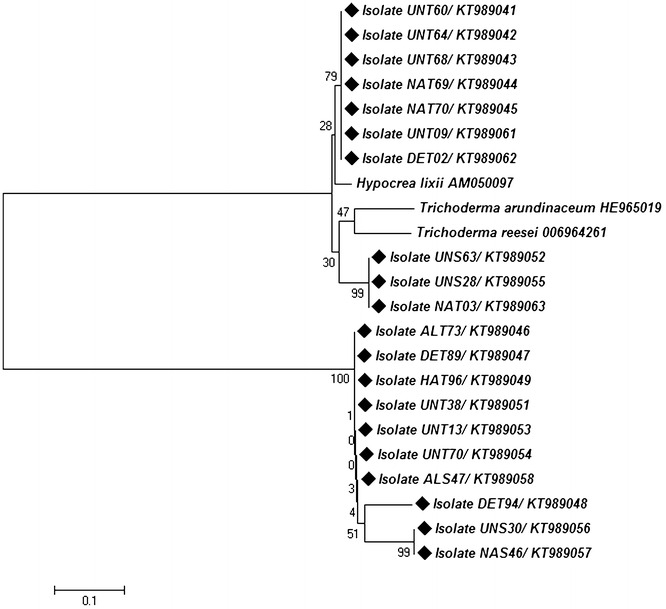
Phylogenetic analysis of the *ERG*1 sequences from different isolates of *Trichoderma* isolates from tomato rhizosphere. Tree was constructed by the neighbour-joining method. The *numbers* given over branches indicate bootstrap coefficient

Detection of squalene epoxidase (*ERG*-1) gene in *Trichoderma* isolates was shown in Fig. 7. Squalene epoxidase (*ERG*-1) gene amplification showed one specific band (500 bp) in all the twenty *Trichoderma* isolates. The phylogenetic tree obtained by sequence analysis of *ERG*1 region of all the tested isolates is represented in Fig. 8. A neighbour-joining analysis of the alienable *ERG*1-sequences of all the tested isolates demonstrated two distinct phylogenetic clades. Clade A comprised mainly *T. harzianum* (UNT60, UNT64, UNT68, NAT69 and UNT70), *T. viride* (UNT09, DET02 and NAT03), *T. koningii* (UNS63) and *T. virens* (UNS28) and showed very high homology to the nearest *ERG*1 sequence of *H. lixii*, *T. arundinaceum* and *T. reesei* submitted in NCBI GenBank. Clade B represented four isolates of *T. harzianum* (ALT73, DET89, HAT96, and DET94), two isolates of *T. koningii* (UNT38), two isolates of *T. asperellum* (UNT13 and UNT70) and three isolates of *T. virens* (ALS47, UNS30 and NAS46) and showed heterogeneity with respect to the *ERG*1 sequence of *H. lixii*, *T. arundinaceum* and *T. reesei.*

## Discussion

Microbial inoculants with antagonistic properties towards fungal plant pathogens have a potential to replace chemical pesticides since they are known for growth promotion and disease reduction in crops. Several species of *Trichoderma* have been used as biological control agents to manage diseases of vegetable and other crops (Solanki et al. 2011; Srivastava et al. 2012; Al-Sadi et al. 2015). In the present study, twenty isolates of *Trichoderma* collected from rhizosphere soil of tomato were phenotypically, biochemically and genetically characterized to identify and screen the most efficient antagonistic against four tomato fungal pathogens (FOL, AA, CG and RS). All the tested isolates grew considerably faster than the fungal pathogens and quickly controlled the pathogens. The ability to grow rapidly gives antagonists an important advantage in competition for space and nutrients with pathogen (Benítez et al. 2004; El_Komy et al. 2015). Nine isolates (UNT68, DET94, HAT96, UNT38, UNS30, DET02, ALS47, UNS28 and UNT09) showed significant per cent mycelium inhibition against the test pathogens. These isolates overgrew and sporulated on the pathogen colonies. In the interaction zone, the mycelia of all the fungal pathogens had abnormal morphology and lysed, which implies the occurrence of strong mycoparasitism. These results are in conformity with previous studies where *Trichoderma* isolates showed high capabilities as versatile biocontrol agents (Trillas et al. 2006; Tondje et al. 2007; de los Santos-Villalobos et al. 2013). Interestingly, the interaction of indigenous *Trichoderma* isolates with four different fungal pathogens resulted in significantly different amounts of pathogen inhibition. For instance, DET94 had very strong inhibitory effect on the growth of FOL, RS and CG pathogens, whereas moderate inhibition effect was recorded in case of AA. These results are consistent with the findings of Markovich and Kononova (2003). They reported that the mycoparasitic capacity of various species and isolates of *Trichoderma* differs. There are several mechanisms involved in *Trichoderma* antagonism, namely, antibiosis whereby the antagonist fungus produces antibiotics, competes for nutrients and mycoparasitism, whereas *Trichoderma* directly attacks the plant pathogen by excreting lytic enzymes such as chitinases and β-1,3 glucanases (Kubicek et al. 2001; Radjacommare et al. 2010; Solanki et al. 2011). Such hydrolytic enzymes partially degrade the pathogen cell wall that leads to parasitization (Howell 2003). Also in the present study antagonistic isolates (UNT68, DET94, HAT96, UNT38, UNS30, DET02, ALS47, UNS28 and UNT09) with the highest levels of enzyme activities showed the strong inhibitory effect on the growth of fungal plant pathogens. Similar observations were made by Howell (2003), wherein the activity of lytic enzymes (chitinases and β-1,3 glucanses) was responsible for lysis of *R. solani* hyphae through digestion of major cell wall components. There was a positive relationship between the antagonistic capacity of the *Trichoderma* isolates and the production of chitinase and β-1,3-glucanases. Thus, efficient antagonistic isolates inhibited fungal growth through the production of lytic enzymes. On parallel lines, Lopes et al. (2012) reported a positive correlation between the lytic enzymes activities and the antagonism capacity of *T. asperellum* against *Sclerotinia sclerotiorum*. Moreover, Qualhato et al. (2013) and El_Komy et al. (2015) reported that there was a positive correlation between the amounts of secreted cell-wall degrading enzymes by *Trichoderma* strains and their ability to control plant pathogenic fungi.

Taxonomic knowledge on *Trichoderma* isolates is important for identification and characterization of potential biocontrol species and to avoid potential risk from introducing an unknown fungal species into the rhizosphere of a given ecosystem. A combination of morphological and molecular methods is desirable for the reliable and accurate identification of *Trichoderma* spp. The few morphological characteristics with limited variation in *Trichoderma* spp. may lead to an overlap and wrong identification of the species (Galarza et al. 2015). In present study, *Trichoderma* isolates were categorized on the basis of description and keys given by Gams and Bissett (1998). As a result, ellipsoidal and sub-globose to globose condial structures resembled with *T. harzianum*, while ellipsoidal and ovoid shaped conidia were matched with *T. virens* isolates; as previously mentioned by Choi et al. (2003). However, some isolates showing overlapping characters and resembling with *T. koningii*, *T. viride* and *T. asperellum* could not be separated using the morphology-based method. Thus, molecular identification of *Trichoderma* isolates at the species level was done on the basis of *TEF*-*1a* gene as it has been reported to be better for distinguishing *Trichoderma* spp. (Samuels 2006).

The present study also revealed the usefulness of DNA polymorphism techniques to detect genetic variation among antagonistic *Trichoderma* isolates. These techniques are important not only for understanding their ecological role in the rhizosphere, but also to characterize the biological control agents for registration and patenting biocontrol strains, recognizing the strains, quality checking during production and ecological characterization (Plimmer 1993). The study of DNA polymorphisms involves the selection of a target sequence, and several approaches have been used to achieve this task. One approach involves the exploitation of ubiquitously conserved known genes that display sequence variation. Identification of *Trichoderma* to the species level based on reference sequences from the National Center for Biotechnology Information correlated with phylogenetic analysis based on sequences of the ITS rRNA and the translation elongation factor gene (*EF*1a). However, the limited intraspecific variation within *Trichoderma* species based on sequences of the *EF1a* gene helped giving better resolution in separating *Trichoderma* species when compared to sequences of the ITS region (Al-Sadi et al. 2015). Thus, in present study, comparative nucleotide sequencing of *EF1a* gene was performed to distinguish and identify antagonistic *Trichoderma* isolates. Based on the sequence analysis of *EF1* gene, the 20 antagonistic isolates were divided in five species: *T. harzianum*, *T. koningii*, *T. asperellum*, *T. virens* and *T. viride*. Another approach involves the screening of random parts of the genome to identify distinctive nucleotide sequences by techniques, such as RAPD, REP-, ERIC- and BOX-PCR. The results indicated that BOX elements and ERIC-PCR are suitable for the rapid genetic differentiation of *Trichoderma* isolates. Some of the *Trichoderma* isolates such as NAT70, UNT64 and ALS47 which were not differentiated by RAPD can be discriminated by BOX and ERIC-PCR banding patterns. In general, both techniques were found to produce reproducible results especially with purified genomic nucleic acid as a template, and when the primer concentration and composition of buffer were strictly controlled. It is also worth mentioning here that ERIC-, REP- and BOX-PCR marker systems revealed >60% intra-species variability among *Trichoderma* isolates, although clustering on the basis of antagonism, geographical origin and hydrolytic enzyme production was not detected. Additionally, the present study was unable to correlate biomarker variation with fungal growth inhibition activity of *Trichoderma* isolates. These findings are in agreement with earlier studies, where no defined correlations between genetic variability assessed by random markers (e.g. RAPD) and the ability of *Trichoderma* isolates to inhibit fungal mycelia growth were obtained (Sharma et al. 2009; El_Komy et al. 2015). This may be due to the ubiquitous nature and seemingly random chromosomal distribution of random repeats in *Trichoderma* genome, giving rise to simultaneous PCR amplification of multiple genomic regions (Rai et al. 2016). The high genotypic variability among *Trichoderma* isolates could be associated with mutations in priming sites, rearrangements of chromosomal segments or recombination process in fungal genomes (Kumar et al. 2012, 2013b). However, genetic variability among *Trichoderma* isolates in addition to their differences in fungal growth inhibition toward fungal plant pathogens suggest that combinations of isolates could further be applied in both greenhouse and field studies to manage tomato diseases.

Terpene compounds (e.g., ergokonins and viridins) are involved in the biocontrol process due to their antifungal properties (Malmierca et al. 2015). Similar to this, the present study also documented the possibility of squalene epoxidase driven triterpene biosynthesis mechanism in biocontrol of tomato wilt and foliar blight diseases. Furthermore,  PCR based detection of *ERG1* gene in antagonistic isolates confirmed the presence of gene at molecular level and Blastn and Blastp results showed the maximum homology with a squalene epoxidase gene. Phylogenetic analysis of squalene epoxidase gene (*ERG*1) sequences revealed close relatedness of *ERG*-1sequences with earlier reported sequences of *H. lixii*, *T. arundinaceum* and *T. reesei.* However, *ERG*1 gene also showed heterogeneity among some antagonistic isolates and it may be possible that squalene epoxidase driven triterpene biosynthesis have an important role in biocontrol mechanisms of tested isolates.

In conclusion, the present study provides preliminary information on the biological control of tomato diseases by correctly identifying the fungal antagonists. Correct identification will provide information on understanding the interparasitic relationship with target pathogens and the subsequent environmental fate of the antagonist needed for effective application. Further, combined studies including biological, biochemical and molecular technologies, are essential to select indigenous antagonistic *Trichoderma* isolates that can be used under different environmental conditions. Genetic variability of squalene epoxidase (*ERG*1) gene among these isolates in addition to their differences in aggressiveness toward multiple fungal pathogens suggest that combinations of isolates could further be applied in both greenhouse and field studies to obtain resistance against multiple fungal pathogens in tomato crop. However, further experiments are needed to validate the role of squalene epoxidase driven triterpene biosynthesis in biocontrol mechanisms of tested isolates.
